# Influenza A virus replicates productively in primary human kidney cells and induces factors and mechanisms related to regulated cell death and renal pathology observed in virus-infected patients

**DOI:** 10.3389/fcimb.2024.1363407

**Published:** 2024-03-25

**Authors:** Benjamin Koch, Mahmoud Shehata, Christin Müller-Ruttloff, Shady A. Gouda, Nils Wetzstein, Sammy Patyna, Anica Scholz, Tobias Schmid, Ursula Dietrich, Christian Münch, John Ziebuhr, Helmut Geiger, Luis Martinez-Sobrido, Patrick C. Baer, Ahmed Mostafa, Stephan Pleschka

**Affiliations:** ^1^ Department of Internal Medicine 4, Nephrology, University Hospital, Goethe University Frankfurt, Frankfurt am Main, Germany; ^2^ Center of Scientific Excellence for Influenza Viruses, National Research Centre (NRC), Cairo, Egypt; ^3^ Institute of Medical Virology, Justus Liebig University Giessen, Giessen, Germany; ^4^ German Center for Infection Research (DZIF), Partner Site Giessen, Giessen, Germany; ^5^ Institute for Biochemistry II, Goethe University Frankfurt, Frankfurt am Main, Germany; ^6^ Department of Internal Medicine 2, Infectious Diseases, Goethe University Frankfurt, Frankfurt am Main, Germany; ^7^ Institute of Biochemistry I, Goethe University Frankfurt, Frankfurt am Main, Germany; ^8^ Institute for Tumor Biology and Experimental Therapy, Georg-Speyer-Haus, Frankfurt am Main, Germany; ^9^ Frankfurt Cancer Institute (FCI), Frankfurt am Main, Germany; ^10^ Cardio-Pulmonary Institute, Frankfurt am Main, Germany; ^11^ Texas Biomedical Research Institute, Disease Intervention & Prevention (DIP) and Host Pathogen Interactions (HPI) Programs, San Antonio, TX, United States

**Keywords:** influenza A virus, acute kidney injury, distal tubular cells, transcriptomics, proteomics, regulated cell death

## Abstract

**Introduction:**

Influenza A virus (IAV) infection can cause the often-lethal acute respiratory distress syndrome (ARDS) of the lung. Concomitantly, acute kidney injury (AKI) is frequently noticed during IAV infection, correlating with an increased mortality. The aim of this study was to elucidate the interaction of IAV with human kidney cells and, thereby, to assess the mechanisms underlying IAV-mediated AKI.

**Methods:**

To investigate IAV effects on nephron cells we performed infectivity assays with human IAV, as well as with human isolates of either low or highly pathogenic avian IAV. Also, transcriptome and proteome analysis of IAV-infected primary human distal tubular kidney cells (DTC) was performed. Furthermore, the DTC transcriptome was compared to existing transcriptomic data from IAV-infected lung and trachea cells.

**Results:**

We demonstrate productive replication of all tested IAV strains on primary and immortalized nephron cells. Comparison of our transcriptome and proteome analysis of H1N1-type IAV-infected human primary distal tubular cells (DTC) with existing data from H1N1-type IAV-infected lung and primary trachea cells revealed enrichment of specific factors responsible for regulated cell death in primary DTC, which could be targeted by specific inhibitors.

**Discussion:**

IAV not only infects, but also productively replicates on different human nephron cells. Importantly, multi-omics analysis revealed regulated cell death as potential contributing factor for the clinically observed kidney pathology in influenza.

## 1 Introduction

Influenza A viruses (IAV) have a single-stranded and segmented RNA genome of negative polarity ((−)ssRNA). In humans, IAV has a predominant tropism for epithelial cells in the respiratory tract (Ibricevic et al., 2006) and is responsible for seasonal local, epidemic, and pandemic outbreaks. Clinical manifestations of IAV infection can vary between aggravated common cold symptoms and severe complications, including a frequently lethal acute respiratory distress syndrome (ARDS). In the United States of America (USA), seasonal human influenza results in approximately 14.5 million medical visits annually (Matias et al., 2016), about 700,000 hospitalizations, and up to 80,000 influenza-related deaths (Rolfes et al., 2018). The influenza case fatality rate (CFR) is usually low (Taubenberger and Morens, 2006), but because of widespread infection, even seasonal influenza-associated respiratory deaths are estimated to account for more than 500,000 deaths per year globally (Iuliano et al., 2018). Infections of a largely immunologically naïve human population with antigenically new IAV variants were associated with an increased CFR. Thus, for example, in 1918, about 500 million people (one-third of the world human population at the time) were infected by an immunogenically new and more pathogenic H1N1 IAV, resulting in an estimated 50–100 million deaths (Johnson and Mueller, 2002; Morens and Taubenberger, 2018).

In humans, the clinical outcome of IAV infections is related to specific features of the viral genome, patient age, pre-existing comorbidities, viral load, and spread to the lower airways and lung, resulting in viral pneumonia (Siefkes et al., 2015). The particular importance of IAV-related respiratory failure (IAV-ARDS) is well known and mainly based on the infection and lysis of alveolar epithelial and endothelial cells, as well as the resulting inflammation, activation of macrophages, hypercytokinemia, and bacterial co- or superinfections (Short et al., 2014). In addition, extrapulmonary complications result in increased morbidity as well as mortality (Lee et al., 2011), and there is evidence to suggest a link to IAV viremia (Kaji et al., 1959; Zhadanov, 1960; Khakpour et al., 1969; Yawn et al., 1971; Jong et al., 2006; Tse et al., 2011).

**Figure 1:**
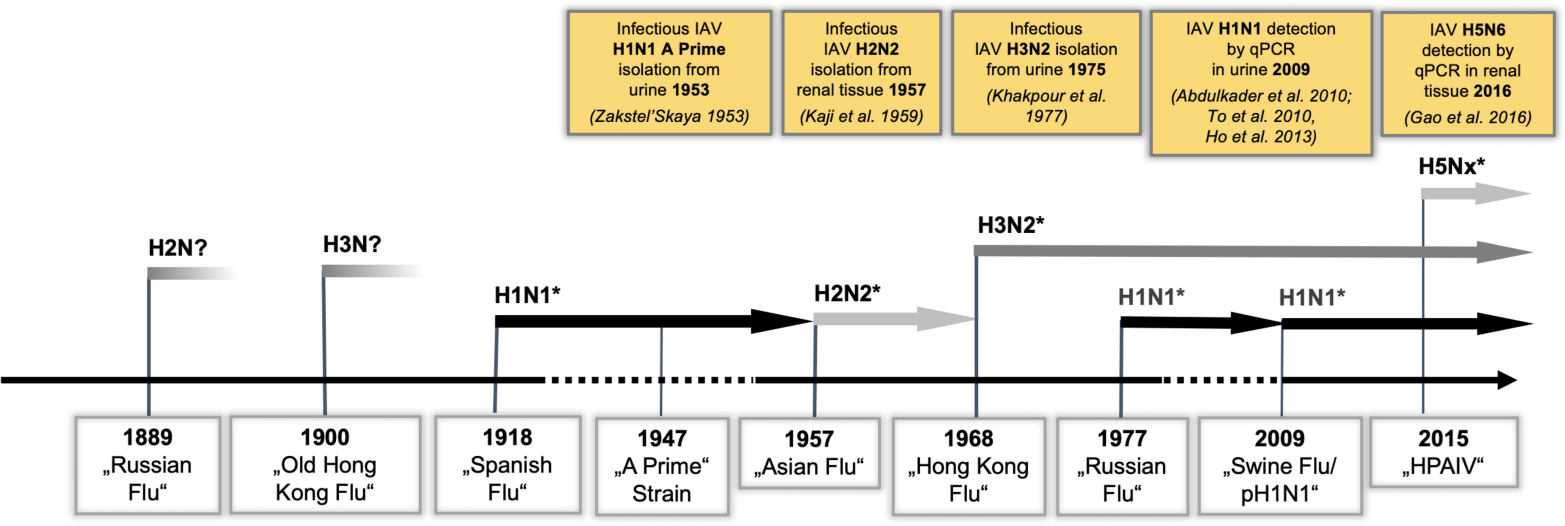
IAV strains detected in renal tissue reflect the dominant circulating strains. IAV pandemic outbreaks and currently cocirculating H1N1, H3N2, and H5Nx strains are indicated. PCR detection or isolation of infectious IAV from renal tissue/urine is illustrated in yellow boxes. (^*^PCR-confirmed IAV strains).

Notably, human and avian IAV receptors are present in human kidney cells (Ulloa and Real, 2001; Yao et al., 2008), and—in addition to liver, spleen, heart, and muscle—infectious IAV has been isolated from kidney tissue in human autopsies (Kaji et al., 1959; Gamboa et al., 1979). Furthermore, IAV could be detected in the urine of patients during several pandemic outbreaks (**Figure 1**) (Zakstel’, 1953; Khakpour and Nik-Akhtar, 1977; Abdulkader et al., 2010; To et al., 2010; Ho et al., 2013). A recent meta-analysis showed that the rate of positive urine tests for IAV was 58% in patients infected with IAV (Lowry et al., 2023). Importantly, the 2009 pandemic was associated with significant extrapulmonary complications (Lee et al., 2011), among which IAV-induced acute kidney injury (IAV-AKI) was related to increased mortality (Abdulkader et al., 2010; Martin-Loeches et al., 2011; Nin et al., 2011a; Thakkar and Golestaneh, 2021), and this trend is continuing in IAV-AKI (Ersoy et al., 2021; Hsu et al., 2022; Jamoussi et al., 2022). Yet, the pathophysiology of IAV-AKI is incompletely understood (Joannidis and Forni, 2011; Watanabe, 2013). Intriguingly, in many lethal cases of human IAV infections, acute tubular necrosis (ATN) of distal tubular cells (DTC) (Beswick and Finlayson, 1959) and viral antigen in DTC and glomerular cells was demonstrated (Zinserling et al., 1983; Nin et al., 2011b). Therefore, the aim of the present study was to assess IAV replication in distinct cells of the kidney, to study alterations of the transcriptome and proteome responses of these cells after IAV infection with the goal of identifying signaling pathway alterations in IAV-AKI, and to compare them to those from IAV-infected lung and primary trachea cells.

## 2 Materials and methods

### 2.1 Cells

Proximal tubular cells (PTC) and DTC were isolated from human kidney tissue provided by Rita Schmitt-Prokopp and Michael Lein (Department of Urology, Sana Hospital, Offenbach, Germany) using magnetic cell separation technology as described previously (Baer et al., 1997). For the isolation of PTC, a monoclonal antibody (mAb) against aminopeptidase N/M (CD13, Cat. No. sc-18899, Santa Cruz, Dallas, Texas, USA, RRID : AB_626895) was used, specific for the proximal tubule. DTC was isolated using a mAb recognizing Tamm Horsfall glycoprotein (THG, clonotype No. 109, purified supernatant from hybridoma provided by Prof. Juergen Scherberich, Munich), a specific antigen of the thick ascending limb of Henle’s loop and the early distal convoluted tubule. DTC was maintained in M199 medium (Sigma-Aldrich, Steinheim, Germany) supplemented with 10% fetal bovine serum (FBS, Biochrom, Berlin, Germany). Conditionally immortalized human glomerular endothelial (CiGEnC, RRID : CVCL_W185), human mesangial (K29-Mes, RRID : CVCL_W168), and human podocyte (Ly8 + 13, RRID : CVCL_W186) cell lines were kindly provided by Simon C. Satchell and Moin A. Saleem (both Bristol Renal, University of Bristol, UK) and maintained according to previously established cell culture protocols (Saleem et al., 2002; Satchell et al., 2006; Sarrab et al., 2011). Briefly, CiGEnC was cultured using EGM-2MV medium + Bullet Kit (No. CC-3202, Lonza, Walkersville, MD, USA). Until 90% confluence, cells were kept at 33°C to allow proliferation. Thereafter, the temperature was switched to 37°C for 10 days to initiate differentiation (all conditions in a 5% CO_2_ atmosphere). K29-Mes and Ly8 + 13 cells were grown at 33°C with RPMI-1640 (ThermoFisher, Germany), 10% FBS, and insulin–transferrin–selenium (ITS, Sigma, No. 1884, final concentration (fconc.) *I* = 5 µg/ml; *T* = 5 µg/ml; *S* = 5ng/ml). After reaching confluence (~80%–90%), they were differentiated at 37°C for 14 days (all conditions in a 5% CO_2_ atmosphere). Madin–Darby canine kidney cells II (MDCK.2, RRID : CVCL_0424) were acquired originally from ATCC and were passaged in DMEM (ThermoFisher, Germany) supplemented with 5%–10% FBS, 100 IU penicillin, and 100 µg streptomycin (P/S)/ml at 37°C in a 5% CO_2_ atmosphere.

### 2.2 Viruses

Human influenza viruses A/Giessen/6/09 H1N1 (H1N1_pdm09_) (Mostafa et al., 2013) and A/Victoria/3/75 H3N2 (H3N2_Victoria_) were obtained from the virus collection of the Institute of Medical Virology, Justus Liebig University Giessen, Germany. The human isolates of a highly pathogenic avian influenza virus (HPAIV), A/Egypt/MOH-NRC-7305/2014 H5N1 (H5N1_MOH-NRC_), and A/Anhui/1/2013 H7N9 (H7N9_Anhui/1_) were kindly provided by M.A. Ali (National Research Center, Cairo, Egypt) and T. Wolff (Robert Koch-Institute, Berlin, Germany), respectively. Stocks of H1N1_pdm09_, H3N2_Victoria_, H5N1_MOH-NRC_, and H7N9_Anhui/1_ were generated on MDCK-II monolayers in the presence of TPCK-treated trypsin as previously described (Pleschka et al., 2016). Briefly, virus stocks were prepared as clarified cell-free supernatants by centrifugation at 800×*g* for 5 min, aliquoted, and stored at −80°C.

To achieve a sufficiently high H1N1_pdm09_ concentration for omics experiments, one-half of the clarified cell-free supernatant of the H1N1_pdm09_ virus was further concentrated by ultracentrifugation as previously described (Pleschka et al., 2016). Briefly, virus particles in the cell-free supernatant of H1N1_pdm09_ were pelleted through a sucrose cushion. A volume of 30 ml of the virus preparation was carefully pipetted on top of a 20% sucrose solution (12 ml) and then centrifuged using a Beckman SW28 rotor at 28,000 r.p.m. for 2 h at 4°C. The pellet was resuspended in 500 µl of PBS. A virus titer of 6 × 10^7^ focus-forming units (FFU)/ml was determined by focus formation assay, and the preparation was stored at −80°C.

### 2.3 Focus formation assay

Virus titers were determined using a focus formation assay as described earlier (Ma et al., 2009). Briefly, 90% confluent MDCK-II cells grown in 96-well plates were washed with PBS++ (PBS containing 1 mM MgCl_2_ and 0.9 mM CaCl_2_). Subsequently, 50 µl of virus-containing supernatant in a 10-fold dilution in PBS/BSA/P/S (PBS++ containing 0.2% BSA; P/S) was added. After 1 h of viral adsorption at room temperature (rt), the virus inoculum was replaced by 150 µl of titration medium (10% of 10× MEM, 33% ddH_2_O, P/S, 1% of 30% BSA, 50% of 1.25% Avicel (DuPont Nutrition Biosciences, Braband, Denmark), 1% DEAE-Dextran, 4% of 7.5% NaHCO_3_, and 2 µg/ml of TPCK-treated trypsin (all tested IAV strains require TPCK-treated trypsin except H5N1_MOH-NRC_). After 24 h postinfection (hpi) at 37°C, the cells were fixed and permeabilized with 330 μl fixing solution (PBS++ containing 4% paraformaldehyde (PFA) and 1% Triton X-100), stored at 4°C for 60 min followed by three washes with PBS/Tween (PBS containing 0.05% Tween 20) and were then incubated for 1 h at rt with 50 µl of first antibody (mouse anti-influenza A nucleoprotein mAb, hybridoma supernatant provided by Prof. Stephan Ludwig, University Muenster, Germany) diluted 1:100 in PBS/BSA (PBS containing 3% BSA). Cells were then washed three times with PBS/Tween and incubated with a second antibody (antimouse HRP antibody, SantaCruz sc2005, RRID : AB_631736) diluted 1:100 in PBS/BSA at rt for 60 min. To detect foci, the washed cells were incubated with 40 µl/well of AEC staining solution (Sigma-Aldrich, USA). For analysis, the 96-well plates were scanned and analyzed using the Photoshop software package (Adobe Systems, San Jose, CA, USA). All titrations were performed in duplicate.

### 2.4 Growth kinetics

PTC, DTC, CiGEnC, CiMes, and CiPod were plated in six-well plates at a density of 4 × 10^5^ cells per well for the infection experiments. Infections with H1N1_pdm09_, H3N2_Victoria_, H5N1_MOH-NRC_, and H7N9_Anhui/1_ were carried out in triplicates at a multiplicity of infection (MOI) of 0.1 and incubated at 37°C in the appropriate medium containing TPCK-treated trypsin (except for H5N1_MOH-NRC_). Cell culture supernatants from infected and control animals were harvested at 12 hpi, 24 hpi, 36 hpi, and 48 hpi. The virus titers were determined by focus formation assay (FFU/ml) using MDCK-II cells.

### 2.5 Infections and sample collection

PTC, DTC, CiGEnC, CiMes, and CiPod were plated in six-well plates at a density of 4 × 10^5^ cells per well. Infections for OMIC analysis were carried out with H1N1_pdm09_ (MOI = 1). For gene expression analysis, infected DTC were collected in RLT+β-mercaptoethanol lysis buffer (Qiagen, Hilden, Germany) at the indicated time points hpi. For mass spectrometry, DTC monolayers were washed twice with PBS++, scratched off, and collected in 1 ml of PBS. The cells were then pelleted by centrifugation for 30 s at 13,000×*g* at 4°C. The supernatant was discarded, and cell pellets were lysed on ice for 10 min using lysis buffer (TLB: 25 mM Tris, pH 8.0; 137 mM NaCl; 10% glycerol; 0.1% sodium dodecyl sulfate; 0.5% sodium deoxycholate; 1% NP-40; 2 mM EDTA, pH 8.0; 0.2 mM pefablock; 5 µg/ml aprotinin; 5 µg/ml leupeptin; 1 mM Na-vanadate; and 5 mM benzamidine). Subsequently, the samples were centrifuged at 13,000×*g* at 4°C for 30 min, and the supernatants were collected for subsequent analyses.

### 2.6 RT-qPCR analysis

Relative expression levels of selected mRNAs were quantified following infection with DTC (MOI = 1). Total RNA was extracted using the RNeasy Maxi Kit (Qiagen, Hilden, Germany), including on-column removal of DNA by digestion with rDNase for 15 min at rt, and cDNA for quantitative real-time RT-PCR (qPCR) was synthesized using 1,000 ng RNA in a 15-μl reaction volume. qPCR reactions were set up to a final volume of 20 μl using the HOT FIREPol^®^ EvaGreen^®^ qPCR Supermix (Solis Biodyne, Tartu, Estonia) and primers for ß-ACTIN (FWD 5′-ACTGGAACGGTGAAGGGTGAC-3′, REV 5′-AGAGAAGTGGGGTGGCTTTT-3′, product size: 169 bp), MLKL (FWD 5′-AAGAAGGTGGAAGAGCGAGC-3′, REV 5′-TCCTTGGTCCTGGAGCATCT-3′, product size: 186 bp), and ZBP1 (FWD 5′-ACCTTCTGGACATGGATGAGCA-3′, REV 5′-AGGCTGACTTTGCTCTTCTTCC-3′, product size: 81 bp). The qPCR reaction was run on an ABI PRISM 7900HT Fast Real-Time PCR System with Sequence Detection System SDS 2.4.1 software (both Applied Biosystems, Waltham, MA, USA), using 40 cycles of the following program: 95°C for 15 s, 63°C for 15 s, and 72°C for 20 s (SDS 2.4.1. settings: automatic baseline, threshold 0.2). To exclude artifacts resulting from primer dimer formation, melting curve analysis was performed using the sequence 95°C for 15 s, 60°C for 15 s, 95°C for 1 min, and 37°C for 30 s. The results shown represent the mean of three independent experiments. Relative expression of the mRNA target was assessed using the ΔΔCt method (Pfaffl, 2001), with β-actin as calibrator, and levels of target gene expression were estimated by 2−ΔΔCt. Statistical significance was determined by GraphPad Prism software version 9.0 for OS X (GraphPad Software Inc., La Jolla, CA, USA) with a two-way ANOVA and *t*-test.

### 2.7 Transcriptome analysis

Six biological replicates of DTC grown in six-well plates were either left uninfected or infected with H1N1_pdm09_ at a MOI = 1. Two replicates of each set were analyzed for transcript expression changes by RNAseq, while the remaining replicates were used for verification of RNAseq results by qPCR (RT-qPCR for ZBP1 and MLKL, **Supplementary Figure S1**). Briefly, total RNA was extracted 12 hpi of the H1N1_pdm09_-infected/noninfected DTC by Qiagen RNeasy Maxi Kit (Qiagen, Germany), including on-column removal of DNA by digestion with rDNase for 15 min at rt. For library preparation, 500 ng of RNA (each showing a RIN score from > 9) was used. Second to depletion of ribosomal RNA (QIAseq FastSelect RNA Removal Kit, Qiagen, Germany), directional libraries were prepared with a NEBNext^®^ UltraTM Directional RNA Library Prep Kit for Illumina^®^ sequencing (No. E7420S, New England Biolabs, Ipswich, MA, USA). Sequencing was performed by an Illumina NextSeq500^®^ using a NextSeq^®^ 500/550 High Output Kit v2 (75 cycles, No. FC-404-2005, Illumina, San Diego, CA, USA). Illumina BCL files were converted to FASTQ by bcl-convert (Illumina, San Diego, CA, USA), quality controlled by FastQC (Babraham Institute, Cambridge, UK, http://www.bioinformatics.babraham.ac.uk/projects/fastqc/) and Illumina adapters trimmed by Trimmomatic v0.40 (Bolger et al., 2014). Quantification of transcript expression was performed by “kallisto” software (Bray et al., 2016) (version 0.46.1) with standard configurations using a kallisto index build from the hg38 assembly (GRCh38.p12 cDNA). “sleuth” (Pimentel et al., 2017) software (version 0.30.0) was used for differential expression analysis with the Wald test setting for comparison of the two groups. Raw (FASTQ), kallisto, and sleuth data are available from NCBI Gene Expression Omnibus (GEO), https://www.ncbi.nlm.nih.gov/geo/query/acc.cgi?acc=GSE189735. DTC transcriptome data were supplemented by existing GEO datasets of IAV-infected cells. The FASTQ datasets (GSE103604, GSE89008) from H1N1_A/PR/8/1934_-infected adenocarcinomic human alveolar basal epithelial (A549) cells (Zhao et al., 2018) as well as from H1N1_A/California/04/09_-infected human bronchial tracheal epithelial (HBTE) cells (Heinz et al., 2018), compared to mock control at 12 hpi, were reanalyzed by kallisto and sleuth software in order to create a homogenous basis for the comparison with the data gained from the H1N1_pdm09_-infected DTCs. All results were investigated for pathway over-representation at the gene level (cut off: at least 2 genes in pathway annotation and *p* < 0.01) by ConsensusPathDB (Kamburov et al., 2011) (http://cpdb.molgen.mpg.de/, Release 35, database = 05.06.2021) employing “Kyoto Encyclopedia of Genes and Genomes (KEGG)” (Kanehisa, 2000), Reactome (Vastrik et al., 2007), and Wikipathways (Pico et al., 2008). GO analysis was performed by ShinyGO v0.77 (Ge et al., 2019). MA plots, heatmaps, and KEGG enrichment/mapping were done by the “R package iDEP” (version 1.0) and “pathview” software (version 1.38) (Luo and Brouwer, 2013; Ge et al., 2018). Network analysis of differentially regulated coding genes using the Ensembl gene set (“ENSG”) was performed by “STRING v11.0” (Mering et al., 2003) software (https://string-db.org) using standard parameters. Next, the network was imported into “Cytoscape 3.8.0” software (https://cytoscape.org) (Shannon et al., 2003), and centrality analysis (top 10 nodes ranked by degree scores) was performed using the “Cytoscape plugin CytoHubba v0.1” (Chin et al., 2014). For Venn (overlap) analysis, BioTools.fr (http://biotools.fr/misc/venny) was utilized.

### 2.8 Sample preparation for LC-MS

Lysates were precipitated using volumes of ice-cold methanol/chloroform/ddH_2_O (3:1:2.5). After centrifugation at 14,000×*g* for 45 min at 4°C, the upper aqueous phase was aspirated, and 3 volumes of ice-cold methanol was added. Samples were mixed, and proteins were pelleted by centrifugation at 14,000×*g* for 5 min at 4°C. The supernatant was discarded, and the pellets were washed one additional time with ice-cold methanol. Protein pellets were dried at rt for further use. Proteins were resuspended in 8 M urea, 10 mM EPPS at pH 8.2, and 1 mM CaCl_2_, and protein concentration was determined using a BCA assay (No. 23235, ThermoFisher Scientific, Waltham, MA, USA). Samples were then diluted to 2 M urea using digestion buffer (10 mM EPPS at pH 8.2, 1 mM CaCl_2_) and incubated with LysC (Wako Chemicals, Osaka, Japan) at a 1:50 (w/w) ratio overnight at 37°C. The next day, digestion reactions were further diluted to 1 M urea using digestion buffer and incubated at a 1:100 (w/w) ratio of trypsin (No. V5113, Promega, Madison, WI, USA) for an additional 6 h at 37°C. Digests were acidified using trifluoroacetic acid (TFA) to a pH of 2–3, and peptides were purified using Sep-Pak C18 columns (No. WAT054955, Waters, Milford, MA, USA) according to the manufacturer’s protocol. The eluates were dried and stored for further processing. Peptides were resuspended in TMT-labeling buffer (0.2 M EPPS at pH 8.2, 10% acetonitrile), and peptide concentration was determined by BCA assay. Peptides were mixed with TMT reagents (No. 90111, No. A37724, No. 90061, ThermoFisher Scientific, USA) in a 1:2 (w/w) ratio (2 mg TMT reagent per 1 mg peptide). Reactions were incubated for 1 h at rt and subsequently quenched by the addition of hydroxylamine to a final concentration of 0.5% at rt for 15 min. Samples were pooled in equimolar ratio, acidified, and dried for further processing. Before MS analysis, peptide samples were purified using Empore C18 (Octadecyl) resin material (3 M Empore, St. Paul, MN, USA). The material was activated by incubation with methanol for 5 min, followed by one wash each with 70% acetonitrile/0.1% TFA and 5% acetonitrile/0.1% TFA. Samples were resuspended in 5% acetonitrile/0.1% TFA and loaded onto the resin material. Peptides were washed with 5% acetonitrile/0.1% TFA and eluted with 70% acetonitrile (ACN). Samples were dried and resuspended in 0.1% formic acid (FA) for LC-MS2/3.

### 2.9 High-pH reverse phase fractionation

Peptides were either fractionated using a Dionex Ultimate 3000 analytical HPLC or a high-pH reversed-phase fractionation kit (ThermoFisher Scientific, USA). The latter was used according to the manufacturer’s instructions. For high pH reversed-phase fractionation on the Dionex HPLC, 500 mg of pooled and purified TMT-labeled samples were resuspended in 10 mM ammonium bicarbonate (ABC), 5% ACN, and separated on a 250-mm-long C18 column (Aeris Peptide XB-C18, 4.6 mm ID, 2.6 mm particle size; Phenomenex, Torrance, CA, USA) using a multistep gradient from 100% solvent A (5% ACN, 10 mM ABC in water) to 60% solvent B (90% ACN, 10 mM ABC in water) over 70 min. Eluting peptides were collected every 45 s into fractions, which were cross-concatenated and dried for further processing.

### 2.10 Mass spectrometry

Peptides were resuspended in 0.1% FA and separated on an Easy nLC 1200 (ThermoFisher Scientific, USA) and a 22-cm-long, 75-mm ID fused-silica column, which had been packed in-house with 1.9 mm C18 particles (ReproSil-Pur, Dr. Maisch, Germany), and kept at 45°C using an integrated column oven (Sonation, Biberach, Germany). Peptides were eluted by a nonlinear gradient from 5% to 38% acetonitrile over 120 min and directly sprayed into a QExactive HF mass spectrometer equipped with a nanoFlex ion source (ThermoFisher Scientific, USA) at a spray voltage of 2.3 kV. Full-scan MS spectra (350–1,400 m/z) were acquired at a resolution of 120,000 at m/z 200, a maximum injection time of 100 ms, and an AGC target value of 33,106. Up to 20 most intense peptides per full scan were isolated using a 1-Th window and fragmented using higher-energy collisional dissociation (normalized collision energy of 35). MS/MS spectra were acquired with a resolution of 45,000 at m/z 200, a maximum injection time of 80 ms, and an AGC target value of 13,105. Ions with charge states of 1 and > 6, as well as ions with unassigned charge states, were not considered for fragmentation. Dynamic exclusion was set to 20 s to minimize repeated sequencing of already-acquired precursors.

### 2.11 Processing of proteomics raw files and data analysis

Raw files were analyzed using Proteome Discoverer (PD) 2.2 software (ThermoFisher Scientific, USA). Files were recalibrated using the Homo sapiens SwissProt database (TaxID:9606, version 2017-06-07). Spectra were selected using default settings, and database searches were performed using the SequestHT node in PD. Database searches were performed against trypsin-digested Homo sapiens SwissProt database and FASTA files of common contaminants (“contaminants.fasta” provided with MaxQuant) for quality control. The results were then exported to Excel files for further processing. Log2 fold changes were calculated by log2 transformation of the ratio between the mean of the replicates of treated samples versus the control samples. Significance was assessed by unpaired, two-sided Student’s *t*-test. *p*-values were adjusted by a Benjamini–Hochberg FDR correction. Adjusted *p*-values (*q*-values) lower than 0.05 were considered significant. *n* represents the number of independent replicates. Data are available via https://www.ebi.ac.uk/pride/archive with identifier PXD030093.

### 2.12 Cell viability and viral inhibition assays

Confluent layers of DTC in 96-well plates were infected with H1N1_pdm09_ or H3N2_Victoria_ at a MOI of 0.1. One hour after infection, emricasan (EM, No. HY-10396, MedChemExpress, Monmouth Junction, NJ, USA) and necrosulfonamide (NSA, No. HY-100573, MedChemExpress, USA) were added at different concentrations (EM: 10 µM, NSA: 5 µM, or EM: 10 µM + NSA 5 µM) and cells incubated at 37°C for 24 hpi in M199 (containing 0.2% bovine serum albumin (BSA) (Sigma, Germany) and 1 µg/ml of TPCK-treated trypsin. Cytopathogenic effects/viability was assessed using CellTox™ Green Cytotoxicity Assay (No. G8741, Promega, USA) and RealTime-Glo™ Extracellular ATP Assay (No. GA5010, Promega, USA). Data for each condition were collected for at least three biological replicates using a Spark 10M multimode microplate reader (Tecan, Zurich, Switzerland). Epithelial protection was calculated as a relative reduction of viability loss compared to uninfected controls. The cytotoxicity of the different inhibitors was determined using the tetrazolium derivate XTT (XTT Cell Viability Kit, Biotium, Hayward, CA, USA) according to the manufacturer’s protocol. Briefly, DTC was seeded in 96-well plates and incubated for 24 h with the agent to be tested at the indicated final concentrations. Next, activated XTT was added, and after 5 h of incubation at 37°C, the absorbance was measured by a microplate reader (LB 911 Apollo-1, Berthold Technologies, Bad Wildbad, Germany) at 450 nm.

### 2.13 Quantification and statistical analysis

Results were expressed as the mean ± standard deviation (SD). Error bars represent the mean ± SD of at least three independent experiments. The difference between the two mean values was analyzed using Dunnett’s multiple comparison test or Student’s *t*-test using GraphPad Prism software version 9.0 for OS X (GraphPad Software Inc., USA). The difference was considered statistically significant when *p* < 0.05. Constitutive gene expression was assumed within a log2 fold change (log2FC) of ± 0.25.

### 2.14 Biosafety

All experiments with infectious viruses were performed according to German regulations for the propagation of IAV. All experiments involving HPAIV were performed in a biosafety level 3 (BSL3) containment laboratory approved for such use by the local authorities (RP, Giessen, Germany).

### 2.15 Ethics approval statement

The authors have no ethical conflicts to disclose. The procurement procedure for human PTC and DTC isolation was approved by the ethics committee of the Goethe-University Hospital Frankfurt, Germany, file number UGO 03/10–4/09.

## 3 Results

### 3.1 Human and avian IAV can infect and replicate in human kidney cells *in vitro*

**Figure 2:**
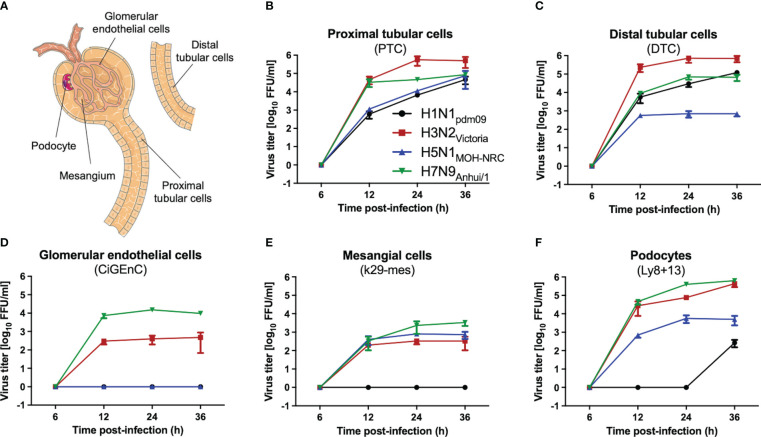
IAV replication on human kidney cells. Human primary (PTC, DTC) or immortalized (K29-Mes, CiGEnC, Ly8 + 13) kidney cells were infected with IAV H1N1_pdm09_, H3N2_Victoria_, H5N1_MOH-NRC_, and H7N9_Anhui/1_, and viral titers were determined by focus-forming assay (FFU/ml) at the indicated time points (error bars indicate standard deviations (SD) of the data obtained from three biological replicates at each time point). **(A)** A renal functional unit (nephron), loop of Henle omitted. **(B)** Human primary proximal tubular cells (PTC). **(C)** Human primary distal tubular cells (DTC). **(D)** Human conditionally immortalized glomerular endothelial cells (CiGEnC). **(E)** Human conditionally immortalized mesangial cells (K29-Mes). **(F)** Human conditionally immortalized podocytes (Ly8 + 13).

To investigate whether IAV can infect and replicate productively in human kidney cells, primary proximal (PTC) and distal tubular cells (DTC), as well as immortalized glomerular endothelial (CiGEnC), mesangial (K29-Mes), and podocyte (Ly8 + 13) human cell lines, were used as an *in vitro* model. Cells were infected with H1N1_pdm09_ and IAV H3N2_Victoria_ human IAVs, as well as with human isolates of either low-pathogenic avian influenza virus (LPAIV) H7N9_Anhui/1_ or highly pathogenic avian influenza virus (HPAIV) H5N1_MOH-NRC_. The analysis of IAV titers in supernatants collected from these infected cells revealed remarkable differences in replication efficiency depending on the cell type and IAV strain used (**Figure 2**). Generally, primary tubular cells (DTC, PTC, **Figure 2A**) allowed replication of all IAV strains (**Figures 2B, C**). However, H5N1_MOH-NRC_ and H1N1_pdm09_ did not replicate in glomerular endothelial cells (CiGEnC, **Figure 2D**), and H1N1_pdm09_ also did not replicate in mesangial cells (K29-Mes, **Figure 2E**) within 36 hpi and only late and with low titers on the podocyte cell line (Ly8 + 13, **Figure 2F**). In contrast to other IAV isolates, H3N2_Victoria_ and H7N9_Anhui/1_ replicated well on all cell types included in this analysis (**Figures 2B–F**). In conclusion, all investigated IAV strains replicated well on primary tubular cells. Given the *in vitro* data, it is possible that the different IAV strains may have different effects on renal pathology *in vivo*.

### 3.2 Transcriptome and proteome analysis of H1N1_pdm09_-infected DTC

**Figure 3:**
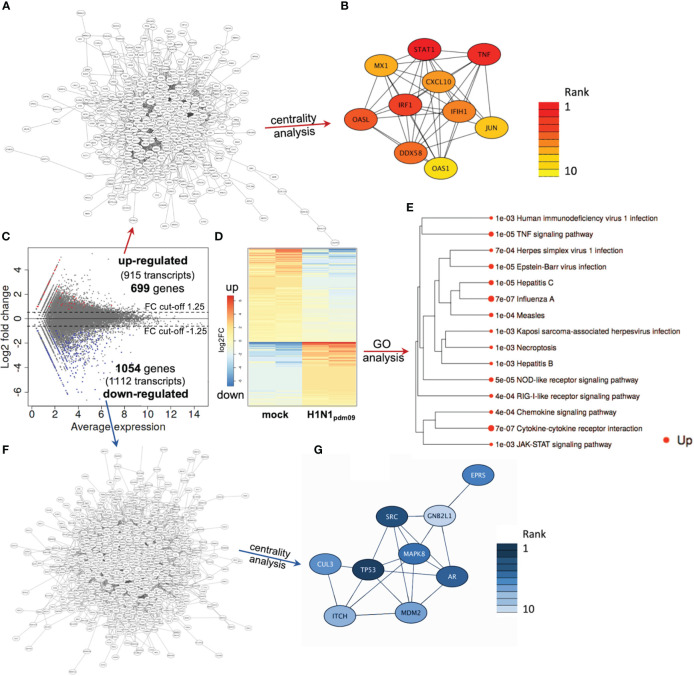
DTC transcriptomics. **(A)** Network of upregulated coding transcripts (587 nodes). **(B)** Network/centrality analysis for top 10 node interactions of proteins coded by upregulated transcripts (colored/dark red represents the highest centrality score). **(C)** MA plot of transcript expression changes 12 h after infection of DTC with H1N1_pdm09_, showing significant changes in red (upregulated) and blue (downregulated). **(D)** Heatmap of differentially expressed genes (H1N1_pdm09_-infected DTC vs. mock DTC, blue = downregulated, red = upregulated). **(E)** Enrichment tree of differentially regulated KEGG pathways from **Figure 3D**. The dot size represents the *q*-value (low *q*-value = large dot). **(F)** Network of downregulated coding transcripts (969 nodes). **(G)** Network/centrality analysis restricted to top 10 node interactions of proteins coded by downregulated transcripts (colored/dark blue represents the highest centrality score).

**Figure 4:**
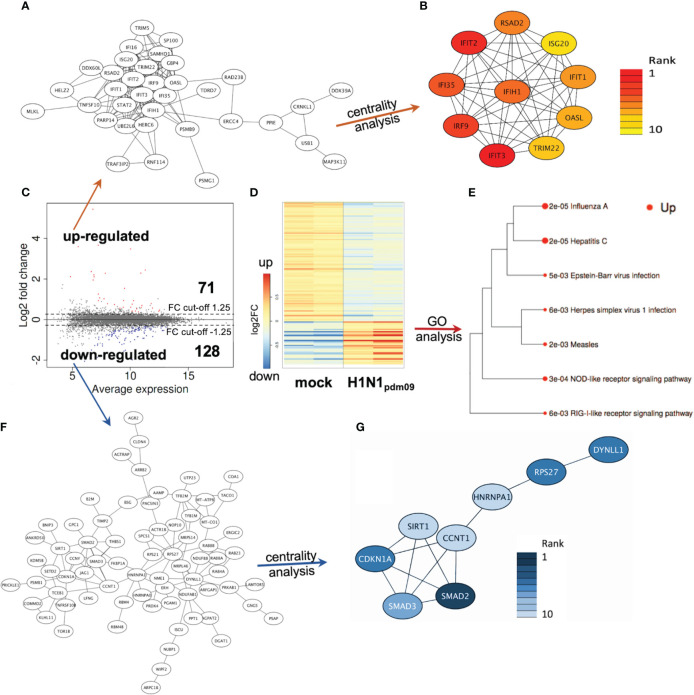
DTC proteomics. **(A)** Network of upregulated proteins (41 nodes). **(B)** Network/centrality analysis for top 10 node interactions of proteins (colored/dark red represents the highest centrality score). **(C)** MA plot of protein changes 12 h after infection of DTC with H1N1_pdm09_, showing significant changes in red (upregulated) and blue (downregulated). **(D)** Heatmap of differentially regulated proteins in H1N1_pdm09_-infected DTC vs. mock DTC (blue = downregulated, red = upregulated). **(E)** Enrichment tree of differentially regulated KEGG pathways from **Figure 5D**. The dot size represents the *q*-value (low *q*-value = large dot). **(F)** Network of downregulated proteins (84 nodes). **(G)** Network/centrality analysis restricted for the top 10 node interactions of downregulated proteins (colored/dark blue represents the highest centrality score).

Based on these initial findings and because of the dominant DTC pathology observed in autopsies of H1N1-infected patients (Kuskow, 1895; Winternitz et al., 1920; Beswick and Finlayson, 1959; Zinserling et al., 1983; Nin et al., 2011a), we subsequently infected DTC for further mechanistical analyses. As IAV infection results in the specific inhibition of cellular mRNA processing and translation (“host-cell shut-off”) (Bercovich-Kinori et al., 2016; Levene and Gaglia, 2018), analyses of both the transcriptome and proteome were performed at 12 hpi (**Supplementary Tables S1**, **S2**). Inoculation of DTC with H1N1_pdm09_ resulted in highly significant transcriptional fold changes (**Figures 3C, D**) and protein abundances (**Figures 4C, D**) as compared to mock-infected DTC. To identify networks between cellular proteins, we employed STRING-DB network analysis. As this is based on protein/protein interactions, noncoding transcripts were excluded, and we restricted this analysis to those affected 1,753 protein-coding transcripts.

#### 3.2.1 Transcriptome and proteome analysis of upregulated genes in H1N1_pdm09_-infected DTC reveals a strong antiviral defense response

Among these 1,753 protein-coding transcripts, the top upregulated ones (**Supplementary Table S1**) are dominantly coding for interferon-induced proteins, like the antiviral-acting IFIT2, the double-stranded RNA sensor IFIH1, the inhibitors of viral endosomal fusion NCOA7 (219 aa isoform) and IFITM3, as well as the viral restriction factor MX1. Network analysis of upregulated protein-encoding transcripts in IAV-infected DTC (699) indicated 587 nodes (**Figure 3A**), of which the top 10 nodes (identified by centrality analysis) all include significantly upregulated transcripts coding for interacting factors involved in antiviral defense (**Figure 3B**). KEGG pathway analysis of differentially regulated genes (**Figure 3D**; **Supplementary Table S3**) highlighted factors known to be enriched in several viral infections—including IAV, as well as NOD-like- and RIG-I-like receptor signaling pathways, necroptosis, and the JAK-STAT signaling pathway (**Figure 3E**). Furthermore, KEGG mapping of upregulated transcripts in IAV-infected DTC highlighted most factors within the known KEGG-pattern of IAV infection (**Supplementary Figure S2**). In conclusion, transcriptome analysis emphasized the activation of IAV infection-specific KEGG-patterns, a strong induction of anti-viral defense, and necroptosis on the transcript level in IAV-infected DTC. As IAV infection results in “host-cell shut-off”, we also analyzed the proteome of H1N1_pdm09_-infected DTC at 12 hpi (**Figure 4**). Interestingly, as with the transcriptome, the proteomic network analysis—based on known interactions/functions and KEGG pathway analysis (**Figure 4E**) of differentially regulated proteins (**Figures 4C, D**)—also demonstrated that the upregulated proteins with the highest fold-change (**Supplementary Table S2**) were related to antiviral defense. Among these proteins are RSAD2, IFIT1, IFIT2, IFIT3, OASL, DDX60L, ISG20, and IRF9 (emphasized by centrality analysis, **Figure 4B**), as well as the MAP kinase MAP3K11 and the ubiquitin ligase HERC6. Taken together, interrogation of both upregulated transcriptome and proteome reveals a strong antiviral defense response in IAV-infected DTC (**Figures 3A–E**, **4A–E**; **Supplementary Tables S1–S3**).

#### 3.2.2 Transcriptome and proteome analysis of downregulated genes in H1N1_pdm09_-infected DTC highlights factors regulated to translation, p53, and TGF-ß signaling

Next, the downregulated transcripts and proteins of IAV-infected DTC were investigated. Among the downregulated protein-coding transcripts, the p53 regulator MDM4, the central player in translation initiation EIF4G3, and the cytokine storm and mortality-associated Poly(rC)-binding protein 2 (PCBP2), known to be targeted by the H5N1 IAV-encoded miR-HA-3p (Li et al., 2018), were found within the top downregulated factors (**Supplementary Table S1**). Additionally, cell proliferation, androgen receptor (AR)-mediated signaling, and ubiquitination were pointed out by network analysis (**Figures 3F, G**). Pathway analysis of downregulated transcripts highlighted signaling by Rho GTPases, NRF2-ARE regulation, and Wnt signaling/pluripotency among the most significantly over-represented pathways (**Supplementary Table S3**). Taken together, transcripts of genes related to inhibitors of cytokine production and inflammation, as well as Rho GTPases, NRF2-ARE regulation, and Wnt signaling/pluripotency are predominant among downregulated transcripts in IAV-infected DTC (based on differential gene expression and pathway analysis). Compared to the number of upregulated proteins (71), the number of downregulated proteins with significant changes was almost double (128), which might be due to the IAV-induced “host-cell shutoff” and/or virus-induced repression of certain factors regulating transcription. Importantly, antiviral-acting proteins like the p53-interacting NME1 (Feng et al., 2018) as well as RPS27 (Li, 2019) were found among the most strongly downregulated proteins (**Supplementary Table S2**). Network analysis (top 10 nodes) underlined IAV-induced negative effects on transcription, translation, SMAD/TGF-ß signaling, cell cycle, intracellular transport, and alternative splicing (**Figure 4G**). Pathway analysis of all downregulated proteins included TGF-beta receptor signaling, endocytosis, FoxO signaling, and energy metabolism (**Supplementary Table S3**). Overall, the analysis of the top downregulated protein-encoding transcripts and expressed proteins revealed strong repression of cellular gene expression regarding antiviral acting proteins, the TP53 network, TGF-ß signaling, ubiquitination, transcription, translation, and alternative splicing.

#### 3.2.3 Coregulated transcripts and proteins in IAV-infected DTC

**Figure 5:**
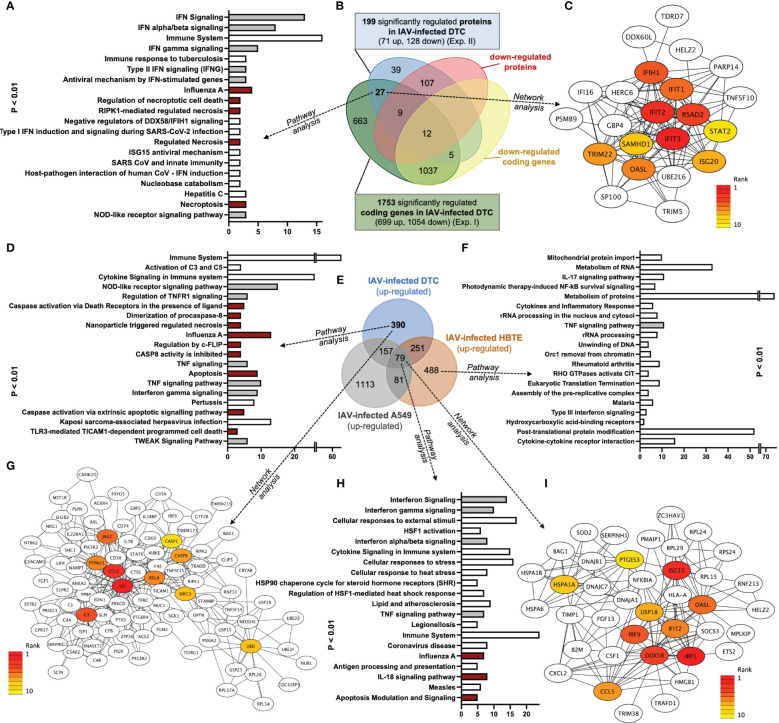
Venn, Network, and GO pathway analyses of common upregulated transcripts and proteins after IAV infection of DTC and upregulated transcripts of IAV-infected DTC, A549, and HBTE cells (dark red depicts pathways directly implicated in PANoptosis; grey shows PANoptosis assisting pathways). **(A)** Pathway analysis of 27 shared upregulated genes (*y*-axis sorted by *p*-value, lowest on top; the *x*-axis shows the count of elements in each pathway). **(B)** Venn analysis of regulated genes in transcriptome and proteome in DTC, separated by up- and downregulation. More proteins (128) and protein-coding transcripts (1,753) are downregulated after IAV infection. **(C)** Core interactions of 27 shared upregulated genes (network/centrality analysis; red represents the highest centrality score). **(D)** Pathway analysis of 390 genes only upregulated in DTC (*y*-axis sorted by *p*-value, lowest on top; the *x*-axis shows the count of elements in each pathway). **(E)** Venn analysis of upregulated genes in DTC, HBTE, and A549 cells 12 h after IAV infection. **(F)** Pathway analysis of 488 genes only upregulated in HBTE (*y*-axis sorted by *p*-value, lowest on top; the *x*-axis shows the count of elements in each pathway). **(G)** Core interactions of proteins coded by 390 only upregulated transcripts in DTC (network/centrality analysis; red represents the highest centrality score). **(H)** Pathway analysis of 79 shared upregulated genes in DTC, HBTE, and A549 cells (*y*-axis sorted by *p*-value, lowest on top; the *x*-axis shows the count of elements in each pathway). **(I)** Core interactions of proteins coded by 79 shared upregulated genes in DTC, HBTE, and A549 cells (network/centrality analysis; red represents the highest centrality score).

Next, we aimed to identify a reliable core set of IAV-induced gene expression changes coinciding with the transcript and protein level in IAV-infected DTC at a late stage of infection (12 hpi). Pathway analysis of 27 mutually upregulated genes and their correlated proteins (**Figures 5A, B**; **Supplementary Table S4**) exposed, especially interferon signaling, members of the IAV infection-related KEGG pattern, regulation of and necroptotic cell death (mixed lineage kinase domain-like pseudokinase (MLKL) as well as TNFSF10 (TRAIL), and ISG15 antiviral mechanism to be significantly over-represented (**Figure 5A**; **Supplementary Table S4**). Furthermore, network analysis highlighted factors of the antiviral defense as core nodes, including interferon response genes like IFIH1, IFIT3, RSAD2, IRF9, OASL, and IFIT1/2 (**Figure 5C**). The shared set of combined downregulated transcripts and proteins (12) analyzed by pathway analysis (**Supplementary Table S4**) pointed out neovascularization processes and Notch signaling. In addition, the shared set of downregulated transcripts and proteins investigated by GO analysis highlights mesenchymal cell differentiation and kidney epithelium development, among others (**Supplementary Table S4**). In conclusion, pathway analysis of coregulated transcripts and proteins revealed induction of especially interferon responses and necroptotic cell death, as well as a downregulation of kidney epithelium development, among others.

### 3.3 Comparison of transcriptomic data from H1N1 IAV-infected DTC, A549, and HBTE cells highlights activation of necroptosis, pyroptosis, and apoptosis

In order to identify a pathology-related gene set dominant in IAV-infected DTC, a comparison with available gene expression omnibus (GEO) data from H1N1-type IAV-infected human A549 alveolar and primary HBTE cells (Heinz et al., 2018; Zhao et al., 2018) was performed (**Figures 5D–F**; **Supplementary Table S5**). Here, we identified 79 shared upregulated (**Figures 5E, H, I**; **Supplementary Table S6**) and 134 shared downregulated genes across all three H1N1 IAV-infected cell lines (**Supplementary Table S6**). Furthermore, the analysis of genes only upregulated in infected DTC revealed members of highly specific pathways (**Figure 5D**; **Supplementary Table S7**), such as activation of complement C3 and C5, the NOD-like receptor (NLR) signaling pathway, regulation of TNFR1 signaling, caspase activation via death receptors, and several pathways involved in regulated cell death, among others (**Figure 5D**; **Supplementary Table S7**). In addition, network analysis of genes only upregulated in infected DTC centered factors important in apoptosis, inflammation, the complement system, and ubiquitination (**Figure 5G**). Furthermore, gasdermin B (GSDMB) and caspase-1 (CASP1), which represent essential factors in pyroptosis (Chen et al., 2018; Tsuchiya et al., 2019; Feng et al., 2022), were upregulated only in infected DTC (**Supplementary Table S6**). However, evidence for regulated cell death was also present in primary human HBTE and A549 cells (**Figures 5E, H**; **Supplementary Table S6**). Although no regulated cell death pathways were among the top 20 pathways (**Figure 5F**), IL-17 signaling was enriched in HBTE cells compared to DTC and A549 cells (**Figure 5F**). Notably, the infection-induced proteotoxic stress response was also among the highly enriched pathways in upregulated genes from IAV-infected DTC, HBTE-, and A549 cells (**Figure 5H**), while network analysis reiterated the ISG15 antiviral response (**Figure 5I**). Crucially, up-regulation of necroptosis and pyroptosis-associated proteins such as MLKL, TNSF10, IRF9, STAT2, and GBP4 were also identified in the proteome analysis of IAV-infected DTC (**Figure 5B**; **Supplementary Figure S3**; **Supplementary Tables S2**, **S3**). For the downregulated gene set in DTC, pathway analysis identified regulation of androgen receptor activity, ephrin signaling, and the RHO GTPase cycle, among others (**Supplementary Table S7**). Taken together, a significant inflammatory response (CCL2, IL6, RELA, JAK2), along with factors of pathogen detection (NLR pathway), upregulation of complement (C3, C5), as well as necroptosis, pyroptosis, and apoptosis indicated by transcripts of pore-forming proteins (MLKL, GSDMD, GSDMB, among others) and caspases (**Figure 5E**; **Supplementary Figures S3**, **S4**; **Supplementary Tables S6**, **S7**) are prominent features of IAV-infected primary DTC.

### 3.4 Inhibition of regulated cell death decreases viral-induced cytopathic effects and viral titer

**Figure 6:**
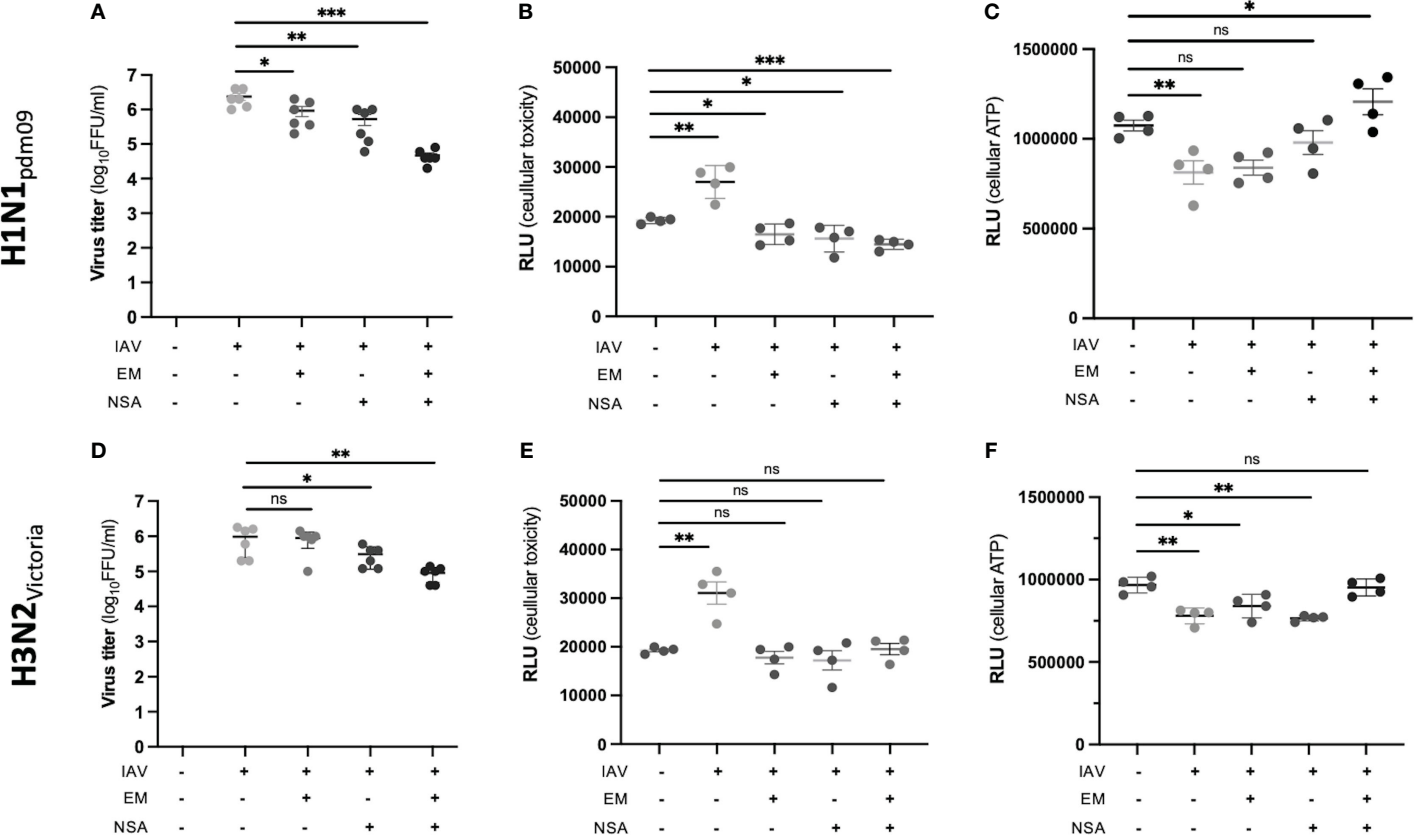
Inhibition of IAV replication and IAV-induced epithelial cell toxicity. EM and NSA inhibitors, alone or in combination, were used to assess their ability to inhibit IAV-induced epithelial cell toxicity (each assay: *n* = 4; ^*^ *p* < 0.05; ^**^ *p* < 0.01; ^***^ *p* < 0.001). **(A)** Viral titer of H1N1_pdm09_-infected human DTC at 24 hpi and the effect of inhibitors. **(B)** Analysis of H1N1_pdm09_-induced cytotoxicity and inhibitor effects. The influx of a DNA dye was measured, indicating the membrane integrity’s state. **(C)** Analysis of H1N1_pdm09_-induced CPE and inhibitor effects, measured by ATP levels, indicating viable cells. **(D)** Viral titer of H3N2_Victoria_-infected DTC at 24 hpi and the effect of inhibitors. **(E)** Analysis of H3N2_Victoria_-induced cytotoxicity and inhibitor effects. The influx of a DNA dye was measured, indicating the membrane integrity’s state. **(F)** Analysis of H3N2_Victoria_-induced CPE and inhibitor effects, measured by ATP levels.

Next, the effect of regulated cell death inhibition in H1N1_pdm09_- and H3N2_Victoria_-infected DTC was investigated (**Figure 6**). To this end, the viral titer as well as the cellular viability with and without emricasan, (EM, a known apoptosis and pyroptosis inhibitor (Natori et al., 2003; Doerflinger et al., 2020)) or/and the necroptosis inhibitor necrosulfonamide (NSA) were studied. The inhibitors alone or in combination had no cytotoxic effect on DTC at the evaluated concentrations (**Supplementary Figure S5**). Notably, both inhibitors resulted in a significant reduction in viral titer at 24 hpi (**Figures 6A, D**), while the most substantial effect was achieved by a combined application of EM and NSA in H1N1_pdm09_-infected DTC. In contrast, their effects in H3N2_Victoria_-infected DTC were less pronounced (**Figures 6A, D**). Furthermore, treatment with EM and NSA preserved cellular integrity, while IAV infection in the absence of the inhibitors resulted in a significant cytopathogenic effect (CPE, **Figures 6B, E**). Accordingly, cellular ATP levels demonstrated stronger tubular epithelial cell protection by the combination of EM and NSA (**Figures 6C, F**).

## 4 Discussion

Here, we demonstrate that human IAV as well as human isolates of avian IAV have the capacity to infect (and propagate in) different human kidney cell types. Furthermore, by transcriptome and proteome analyses, we revealed induction of regulated cell death in H1N1_pdm09_-infected primary human DTC, correlating with the observed kidney pathology in autopsies taken from deceased IAV patients. Up to now, IAV-AKI is not entirely understood. Systemic inflammation, shock, and rhabdomyolysis have been proposed to be the main drivers for AKI and ATN in influenza (Joannidis and Forni, 2011; Watanabe, 2013). Yet, several significant observations have been made, for example: (i) the first detailed ATN description in autopsies of influenza victims dating back to 1895 (Kuskow, 1895); (ii) reports on pathologic swelling of tubular kidney epithelia and acute necrosis of especially DTC in human autopsies (Kuskow, 1895; Lucke et al., 1919; Winternitz et al., 1920); (iii) the isolation of infectious IAV from human renal tissue (Kaji et al., 1959) and urine in the twentieth century (Zakstel’, 1953; Zhadanov, 1960; Khakpour and Nik-Akhtar, 1977) or, more recently, the detection of viral RNA (by RT-PCR) in urine (Abdulkader et al., 2010; To et al., 2010; Ho et al., 2013) and kidney tissue (Gao et al., 2016) of IAV-infected patients; (iv) the detection of viral antigen (by serum-based immunofluorescence) in necrotic DTC from a large autopsy cohort in 1983 (Zinserling et al., 1983); and (v) the detection of IAV nucleoprotein in DTC (autopsies from 2009) indicative of an ongoing IAV replication in these cells (Nin et al., 2011a). In addition to the tubular lumen, a recent study has demonstrated the susceptibility of DTC to IAV infection from a simulated microcirculation at the basal area (Huangfu et al., 2023). Considering the diameter of IAV (80–120 nm (Noda, 2012) and the renal slit diaphragm width of 20–50 nm (Ruotsalainen et al., 1999), it is therefore plausible that IAV might disseminate to DTC via peritubular capillaries.

In our analyses, all used IAV-infected DTC and PTC demonstrated productive viral replication, while endothelial and glomerular cells displayed strain-dependent results. In IAV-infected DTC transcripts related to necroptosis, apoptosis and pyroptosis were enriched in comparison to the transcriptome of A549 and HBTE cells. Regulated cell death has been identified as a crucial factor in AKI (Linkermann et al., 2014; Miao et al., 2019; Priante et al., 2019) but is also implicated in lung injury in fatal IAV infection (Sauler et al., 2018; Faust and Mangalmurti, 2020). These results, revealing upregulation of factors important in pyroptosis, apoptosis, and necroptosis, could be integrated into the recently proposed model of pyroptosis, apoptosis, and necroptosis (PANoptosis) (Samir et al., 2020; Place et al., 2021). Accordingly, infection or cellular stressors can lead to cell death by the combinatory crosstalk of pyroptotic, apoptotic, and necroptotic molecules (like ZBP1, RIPK1, RIPK3, CASP1, CASP8, and NLRP3, MLKL, and GSMDs) to effectively control pathogenic invasion (Christgen et al., 2020). However, there is also the risk of an excessive inflammatory response, resulting in impaired crucial tissue functions (Balachandran and Rall, 2020). Therefore, we investigated the effects of regulated cell death inhibition in IAV-infected DTC by the pan-caspase inhibitor EM (Hoglen et al., 2004) and the necroptosis inhibitor NSA. The combination of these inhibitors has previously been studied only in IAV-infected monocytes, showing monocyte protection only when used in combination, while effects on epithelial cells or viral titers were not reported (Lee et al., 2019). In our experiments, both EM and NSA resulted in a significant reduction in viral titer at 24 hpi, which was potentiated by the combined application of EM and NSA in H1N1_pdm09_-infected DTC. Importantly, the analysis of tubular viability indicated a preservation of cellular integrity by EM or NSA in H1N1-type and H3N2-type infected DTC.

Regarding the analysis of the downregulated patterns in DTC (shared between transcriptome and proteome), we could detect Notch signaling, while androgen receptor (AR) and Ephrin signaling were enriched for downregulated transcripts in H1N1_pdm09_-infected DTC in comparison to H1N1 IAV-infected HBTE or A549 cells. Notably, AR signaling limits lung inflammation in an IAV mouse model (Steeg et al., 2020), whereas Ephrin signaling is important for tubular cell cytoarchitecture and survival (Ogawa et al., 2006; Weiss and Kispert, 2016). Moreover, Notch is necessary for the formation of all nephron segments in renal organogenesis (Chung et al., 2017), and alveolar regeneration by epithelial progenitor cells requires Notch to initiate repair (Vaughan et al., 2015). Therefore, it is conceivable that kidney epithelia regeneration is impaired in IAV-infected DTC.

However, this study has limitations. Because we wanted to closely mimic the human environment, the main experiments were done with primary human instead of mouse or canine cells. Thus, we simulated the epithelial aspect of a human tubule, but the effects of pyroptosis/apoptosis inhibition on other cell types than IAV-infected renal cells need to be studied, as other outcomes on virus replication and cellular survival cannot be ruled out. Moreover, as the passage number for primary DTC is limited before they become senescent and the dilution factor is only 1:3, enough primary DTC for either transcriptomic or proteomic analysis could only be gained from two subsequent cell passages. Therefore, we could only relate the shared up- and downregulated transcript and protein levels from two subsequent experiments. Nevertheless, the IAV-induced host-cell shut-off (which is known to target transcription and translation) (Vreede and Fodor, 2010) was reflected in the DTC transcriptome and proteome analysis of these two experiments by the significantly higher number of downregulated transcripts and proteins at 12 hpi. The discrepancy between the number and types of downregulated proteins and the number and types of downregulated transcripts might be explained by posttranslational protein modifications.

In conclusion, IAV can productively replicate on human primary and immortalized nephron cells, and our multiomics analysis of IAV-infected primary human DTC revealed enrichment of multiple programmed cell death pathways (pyroptosis, apoptosis, and necroptosis) with high relevance for IAV- and AKI-related pathology, correlating with the observed kidney pathology in autopsies taken from deceased IAV patients. Consequently, it seems likely that besides classically approved causes like systemic inflammation, shock, and rhabdomyolysis, direct IAV infection of tubular cells can contribute to ATN in IAV-AKI.

## Data availability statement

The datasets presented in this study can be found in online repositories. The names of the repository/repositories and accession number(s) can be found below: https://www.ncbi.nlm.nih.gov/geo/, GSE189735 https://www.ebi.ac.uk/pride/archive, PXD030093.

## Ethics statement

The studies involving humans were approved by Ethics Committee of the Goethe-University Hospital Frankfurt, Germany. The studies were conducted in accordance with the local legislation and institutional requirements. The human samples used in this study were acquired from primarily isolated as part of your previous study for which ethical approval was obtained. Written informed consent for participation was not required from the participants or the participants’ legal guardians/next of kin in accordance with the national legislation and institutional requirements.

## Author contributions

BK: Writing – review & editing, Methodology, Writing – original draft, Visualization, Investigation, Funding acquisition, Formal analysis, Conceptualization. MS: Writing – review & editing, Writing – original draft, Visualization, Investigation, Formal analysis. CM-R: Writing – review & editing, Writing – original draft, Investigation, Formal analysis. SG: Writing – review & editing, Visualization, Writing – original draft, Investigation, Formal analysis. NW: Writing – review & editing, Writing – original draft. SP: Writing – review & editing, Writing – original draft. AS: Writing – review & editing, Formal analysis, Writing – original draft. TS: Writing – review & editing, Writing – original draft, Resources. UD: Writing – review & editing, Writing – original draft, Conceptualization. CM: Writing – review & editing, Writing – original draft, Resources, Formal analysis. JZ: Resources, Writing – review & editing, Writing – original draft. HG: Writing – review & editing, Writing – original draft, Resources. LM-S: Writing – review & editing, Writing – original draft. PB: Writing – review & editing, Writing – original draft, Resources. AM: Writing – review & editing, Writing – original draft, Visualization, Investigation, Funding acquisition, Formal analysis. StP: Writing – review & editing, Writing – original draft, Supervision, Resources, Funding acquisition, Conceptualization.
