# Supplementary material for: Influenza A virus replicates productively in primary human kidney cells and induces factors and mechanisms related to regulated cell death and renal pathology observed in virus-infected patients
Source: Front Cell Infect Microbiol. 2024 Mar 25;14:1363407. doi: 10.3389/fcimb.2024.1363407 (PMC10999593; doi:10.3389/fcimb.2024.1363407)
Supplement: Supplementary file 1 [file DataSheet_1.pdf]

Supplementary Figures

Supplemental Fig. S1: RT-qPCR results for ZBP1 and MLKL in mock- vs. IAV $H1N1_{pdm09}$-infected DTC.
Supplemental Fig. S2: KEGG IAV-pathway (hsa05164) representation of differentially expressed genes after $H1N1_{pdm09}$ infection of DTC.
Supplemental Fig. S3: KEGG necroptosis pathway (hsa04217) representation of differentially expressed transcripts and proteins after $H1N1_{pdm09}$ infection of DTC.
Supplemental Fig. S4: Regulated cell death factors in DTC.
Supplemental Fig. S5: Inhibitor cytotoxicity in DTC.

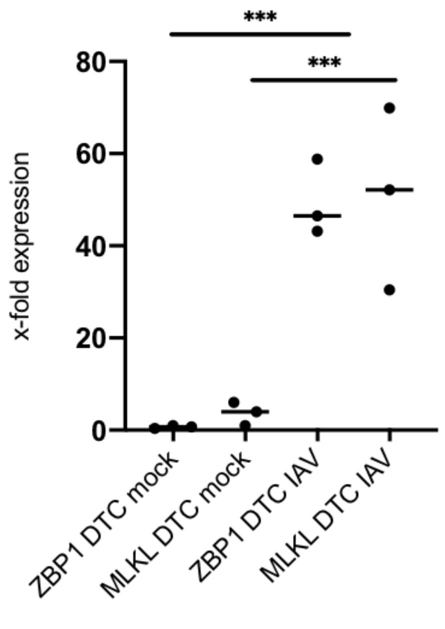

Supplemental Fig. S1: Quantitative real-time PCR results. Analysis of ZBP1 and MLKL in mock- vs. IAV $H1N1_{pdm09}$-infected DTC.

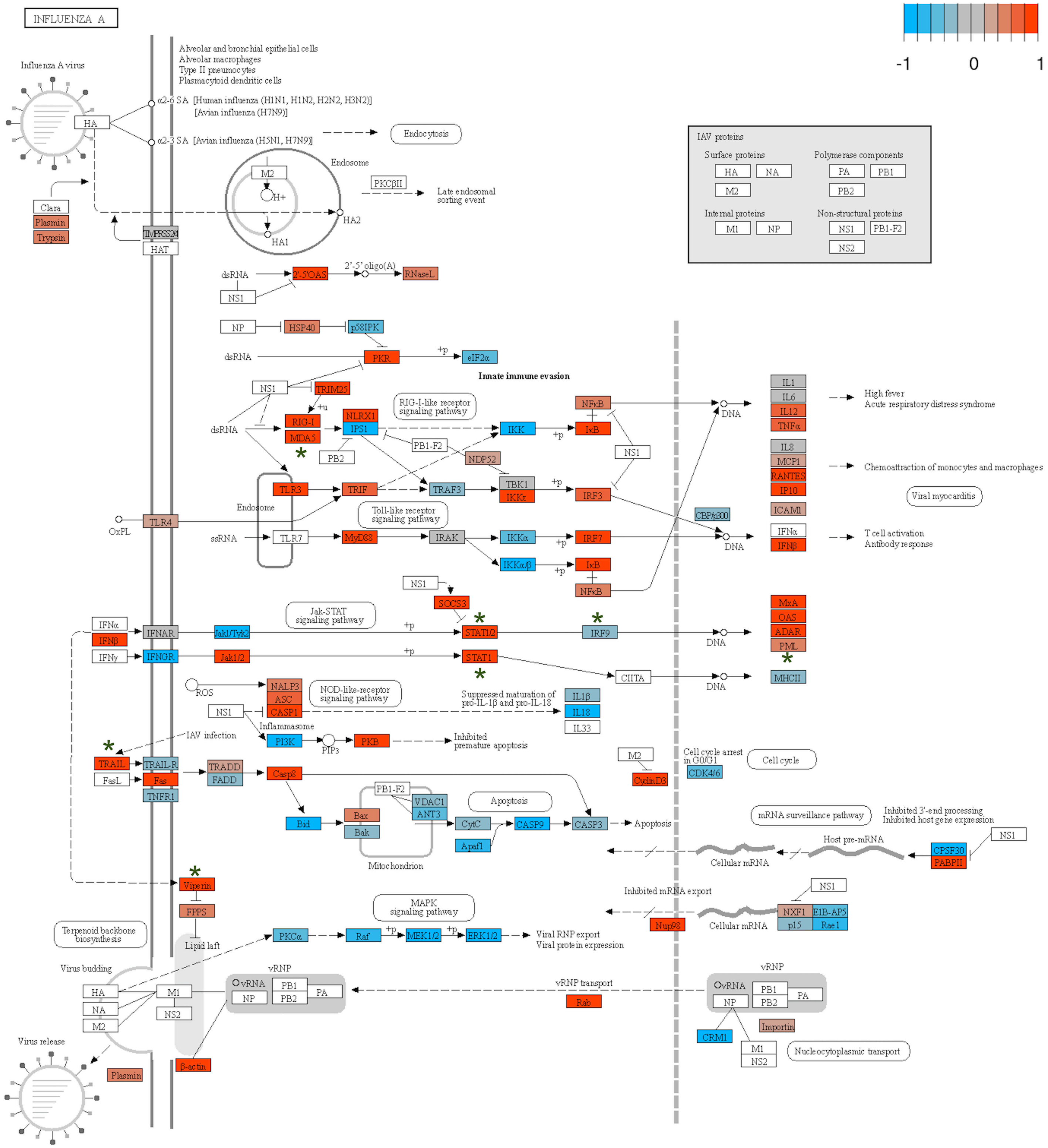

Supplemental Fig. S2: KEGG IAV-pathway (hsa05164) **representation of differentially expressed genes after H1N1$_{pdm09}$ infection of DTC.** The expression change is color coded from red (up-regulated) to blue (down-regulated). The green asterix indicates proteins also represented in the mass spectrometry data.

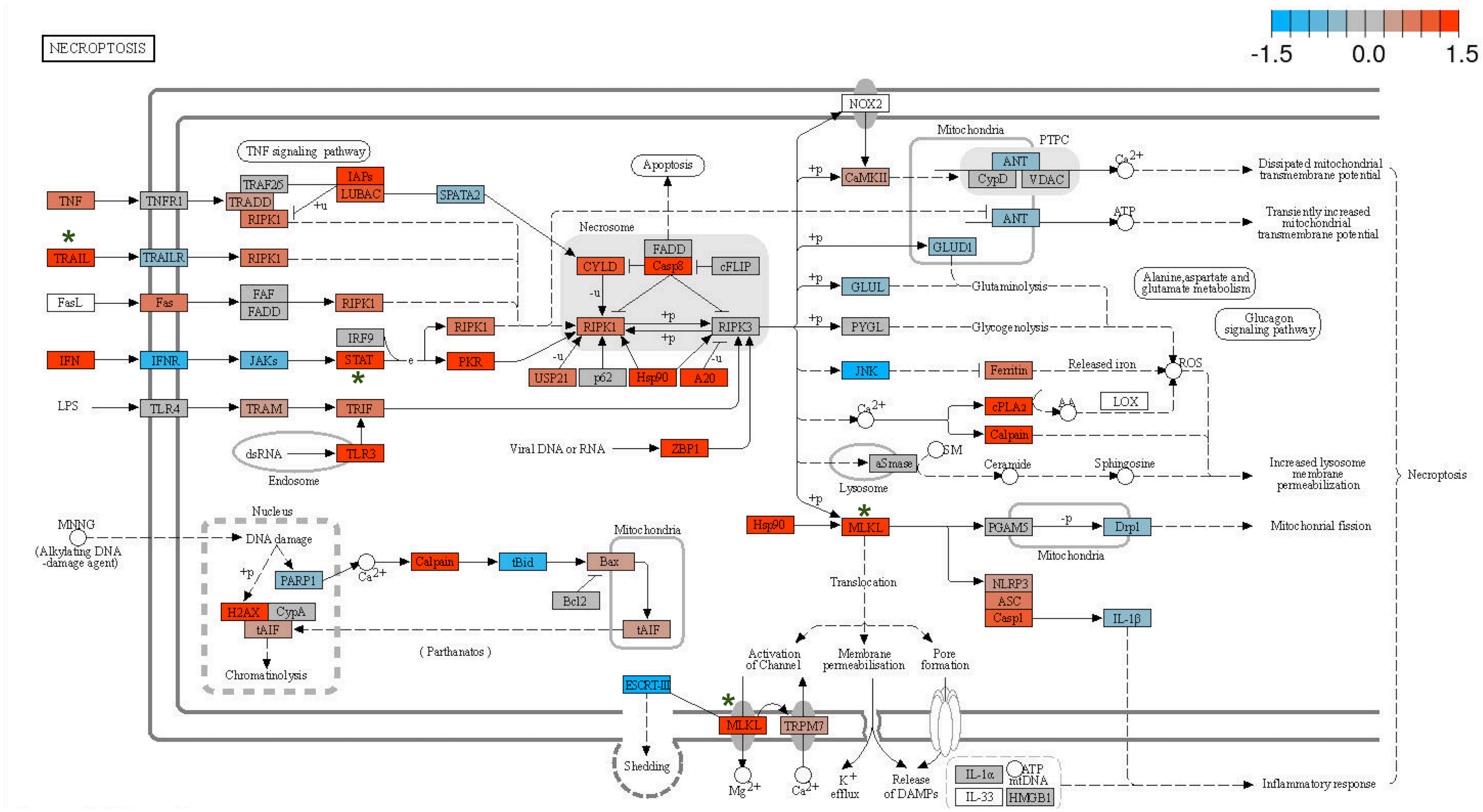

Supplemental Fig. S3: KEGG necroptosis pathway (hsa04217) **representation of differentially expressed transcripts and proteins after H1N1$_{pdm09}$ infection of DTC.** The expression change is color coded from red (up-regulated) to blue (down-regulated). The green asterix indicates genes also represented in the mass spectrometry data.

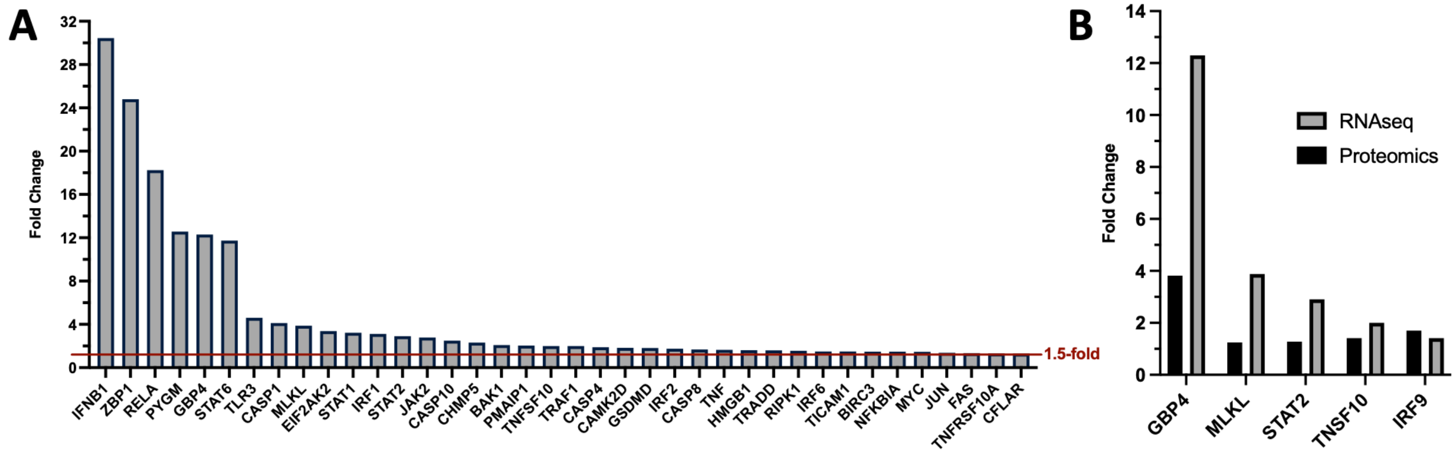

Supplemental Fig. S4: Regulated cell death factors in DTC (as determined by pathway analysis)**. A.** Transcriptomics results (sorted by fold change, all q <0.05), the red-line depicts a 1.5-fold change **B.** Selected regulated cell death factors in transcriptomics and proteomics (sorted by transcriptomics fold change, all p/q <0.05).

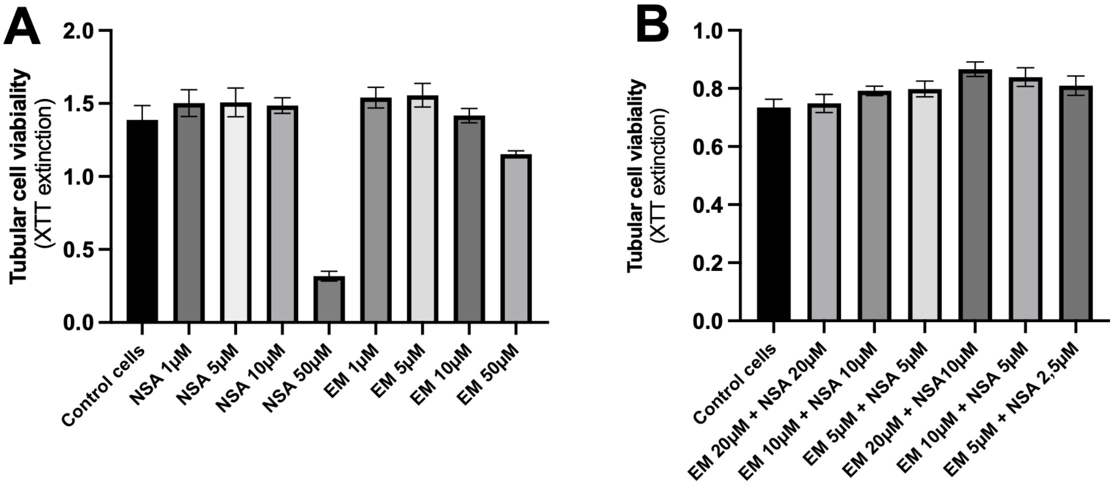

Supplemental Fig. S5: Inhibitor cytotoxicity in DTC. (A) Cytotoxicity of EM respectively NSA analyzed by XTT-assay (NSA 1-50 µM, EM 1-50 µM). **(B)** Cytotoxicity of EM + NSA in different combinations, as analyzed by XTT-assay (EM 5 µM + NSA 2,5 µM to EM 20 µM + NSA 20 µM).
